# Epidemiological and Clinical Risk Factors for Breast Cancer‐Related Lymphedema: A Case‐Control Study From Hamadan, Western Iran

**DOI:** 10.1155/bmri/8099570

**Published:** 2026-09-15

**Authors:** Soulmaz Rahbar, Hojjat Radinmehr, Mohammad Reza Asadi, Tayebeh Roghani, Azade Tabatabaei, Abdolazim Sedighi Pashaki, Erfan Ayubi

**Affiliations:** ^1^ Department of Physiotherapy, School of Rehabilitation Sciences, Hamadan University of Medical Sciences, Hamadan, Iran, umsha.ac.ir; ^2^ Department of Physical Therapy, School of Rehabilitation Sciences, Isfahan University of Medical Sciences, Isfahan, Iran, mui.ac.ir; ^3^ Department of Physiotherapy, School of Rehabilitation, Tehran University of Medical Sciences, Tehran, Iran, tums.ac.ir; ^4^ Cancer Research Center, Institute of Cancer, Hamadan University of Medical Sciences, Hamadan, Iran, umsha.ac.ir

**Keywords:** breast cancer-related lymphedema, case control study, Iran, risk factors

## Abstract

**Background:**

Breast cancer‐related lymphedema (BCRL) is one of main issues following treatments in patients with breast cancer (BC). The current study is aimed at evaluating epidemiological and clinical risk factors for BCRL in female patients with BC.

**Methods:**

In a case‐control study design from October 2024 to March 2025, female BC patients with and without BCRL were recruited from the Lymphedema Physiotherapy Clinic and the Cancer Therapeutic and Diagnosis Imaging Center in Hamadan, Western Iran. Patient, tumor, and treatment characteristics were gathered via interview and reviewing medical and pathology records.

**Results:**

Overall, 230 patients (127 cases and 103 controls) were included. The multivariable analysis revealed several potential risk factors for BCRL: older age at diagnosis (odds ratio [OR] = 1.05; 95% confidence interval [CI], 1.00, 1.10), urban residency (4.76; 1.20, 20.00), axillary lymph node dissection number of 6–9 compared to ≤ 5 (2.54; 0.99, 6.49), pathology grade of 2 (7.11; 1.47, 34.27) and 3 (6.53; 1.32, 32.32) compared to Grade 1, mastectomy versus lumpectomy (2.86; 1.23, 6.67), receiving chemotherapy (9.55; 1.23, 73.95), and positive progesterone receptor (PR) status (2.56; 1.16, 5.55).

**Conclusion:**

The combination of patient, tumor, and treatment characteristics may be associated with BCRL, highlighting the close monitoring of high‐risk patients (older age, high‐grade tumors, mastectomy, and PR‐positive) in Western Iran.

## 1. Introduction

Globally, female breast cancer (BC) is second in new case numbers among all cancers and ranks fourth in the number of deaths [1, 2]. In 2020, over 2.3 million new cases and 685,000 deaths from female BC were reported worldwide. However, the cancer burden projections show that the incident cases and deaths will exceed 3 million and 1 million, respectively, by 2040 [3]. Therefore, early diagnosis, immediate start of treatment, and improving survival are pivotal aspects in the management of female BC.

A delayed diagnosis of the BC can necessitate the application of more aggressive treatment procedures, which can result in complications such as lymphedema [4, 5]. Breast cancer‐related lymphedema (BCRL) is a chronic condition resulting from impaired lymphatic drainage, so protein‐rich lymphatic fluid abnormally accumulates in the interstitial tissues, and limbs become swollen [6]. Pain, redness, tightness, heaviness, paresthesia, fibrosis, and impaired function are common symptoms and complications due to swelling in the limbs. BCRL can along with psychological and physical consequences, which can reduce patients′ quality of life [7]. On the other hand, prospective surveillance programs with prompt intervention can reduce risk of BCRL [8].

Although treatment‐related factors are suggested as leading risk factors for the BCRL, there was evidence regarding the role of demographic, anthropometric, physiological, and biochemical factors in the development of BCRL [9–11]. There is still controversy on well‐established risk factors of the BCRL in terms of direction and strength of association because the results of previous clinical prediction models are function of study population, candidate predictors, and type of model development [11]. In other words, the risk factors of BCRL may vary across different populations. Developing clinical prediction models in independent populations can guide interventions within those populations effectively.

In Iran, BC is first rank in incidence and mortality rates among women [1, 2]. Female BC is one of the three cancers that has seen the greatest increase in recent years [12]. There is spatial inequality in the distribution of female BC within Iran and the trend in incidence and mortality rates varies across the provinces of Iran. For example, Hamadan province was among those with a higher percentage change in incidence rates [13]. Although the epidemiology of female BC is well understood, there is still a lack of knowledge regarding other important aspects, such as complications following BC treatments, for example, BCRL [14]. Considering the above issues, the current study is aimed at evaluating epidemiological and clinical risk factors for BCRL in female patients with BC in Hamadan, Western Iran.

## 2. Methods

### 2.1. Design and Patient

In this case‐control study, female patients with BC were included from October 2024 to March 2025. Patients aged 18 and over and with surgical history were enrolled into the study after obtaining informed consent. Patients with a history of trauma or limb surgery, as well as those with conditions that can cause edema or swelling such as myocardial infarction, renal disease, or gastrointestinal disorders were excluded. Female BC patients with BCRL, as the case group, were selected from those referred to the lymphedema physiotherapy clinic affiliated with the Rehabilitation Faculty, Hamadan University of Medical Sciences. Those without BCRL, as control group, were selected from patients who had visited the Mahdieh Therapeutic and Diagnosis Imaging Center in Hamadan for therapeutic process. All patients were female BC survivors, defined according to the National Cancer Institute as individuals from the time of cancer diagnosis through the remainder of life [15]. Patients who were alive and available for interview during the study period were included, and clinical data were extracted from medical records.

Circumferential assessment of both the affected and unaffected upper limbs was conducted at seven standardized anatomical landmarks. These landmarks included Point 1, located at the base of the fingers along the little finger; Point 2, at the base of the thumb; Point 3, at the wrist; Point 4, positioned midway between the wrist and the elbow crease; Point 5, at the elbow crease; Point 6, located halfway between the elbow crease and the axillary fold; and Point 7, situated 2 cm distal to the axillary fold. To identify the presence of lymphedema, circumferential measurements of the affected limb were compared with those of the contralateral unaffected limb by narrow tape. Lymphedema was diagnosed when the interlimb difference exceeded 2 cm or was ≥ 2 cm at any single measurement site, or when the cumulative difference across all measurement sites exceeded 5 cm or was ≥ 5 cm [16]. The stage of BCRL was defined according to the International Society of Lymphology (ISL) guidelines [17].

To determine the sample size, the assumed proportion of mastectomy in the BC without BCRL was considered 52% based on previous study [14]. Considering a minimum detectable odds ratio (OR) of at least 2.25, two‐sided confidence interval (CI) of 95% (*α* = 0.05), a power of 80% (*β* = 0.20), and a case‐to‐control ratio of 1, the minimum sample size was estimated to be 105 per group.

### 2.2. Data Collection

Patient characteristics, including age at the time of BC diagnosis, marital status, education level, place of residence, type of insurance coverage, number of children, smoking status, family history of BC, and comorbidity with other chronic diseases, were gathered via interview with the patients. Height was measured in a standing position (e.g., without shoes, upright, feet together, heels, and shoulders) against the wall using a tape meter. Weight was measured using a digital electronic weighing scale while wearing minimal clothing and without shoes. Tumor and treatment characteristics were extracted from medical and pathology records. These characteristics involved tumor size, number of axillary lymph node dissection (ALND), number of involved lymph nodes, metastasis, pathological grade, status of hormone receptors, type of surgery, chemotherapy, and radiotherapy.

### 2.3. Statistical Analysis

The characteristics were compared between the two study groups using independent *t* test and chi square test, as appropriate. Variables with a *p* value ≤ 0.10 in univariate analysis were considered potential predictors and were retained for multivariable analysis, in line with the recommendations of Steyerberg and Van Calster [18], who demonstrated that using conventional low *p* value thresholds (e.g., 0.05) for variable selection in prognostic models leads to testimation bias, the exaggeration of effect estimates. The multivariable logistic regression analysis was performed to estimate the adjusted associations. A *p* value ≤ 0.05 was considered as statistical significance in the multivariable analysis. The results were presented as OR with 95% CI. The discriminatory ability of the multivariable analysis was assessed using the area under the receiver operating characteristic curve (AUC‐ROC). As a limited sample size is expected in the combination of risk factors and BCRL levels, a sensitivity analysis using penalized logistic regression via data augmentation with a plausible prior interval for the true effect (OR between 1/40 and 40) was performed to shrink unstable estimates and reduce bias due to sparse data [19]. All statistical analyses were performed using Stata software Version 14.

## 3. Results

Overall, 230 patients (127 cases, 103 controls) were included in the analysis. The median (Quartile 1–Quartile 3) time from surgery to study enrollment of cases and controls was 30 (21–50) and 17 (9–50) months, respectively. Among patients with BCRL, the median time from surgery to lymphedema onset was 11 months (Quartile 1–Quartile 3: 5–25 months; range: 1–72 months), and the median lymphedema duration was 16 months (Quartile 1–Quartile 3: 5–32 months; range: 1–72 months). The left arm was the most affected side (68, 53.5%), and most patients had Grade 2 lymphedema (70, 55.12%). Regarding observed signs, the most frequent sign was fibrosis (50, 39.37%), and 61 patients (48.03%) had no observable signs. Concerning treatment received for BCRL, the most common treatments were massage (45, 35.43%) and lymphotherapy (17, 13.39%). Additionally, 24 patients (18.90%) received complementary and alternative medicine, including leech therapy and cupping, whereas 35 patients (27.56%) received no treatment (Table 1).

**Table 1:** Clinical characteristics of patients with breast cancer‐related lymphedema (*n* = 127).

Characteristic	Value
Time from surgery to study enrollment	30 (21–59); 4–79
Time from surgery to lymphedema onset (months)^a^	11 (5–25); 1–72
Lymphedema duration (months)^a^	16 (5–32); 1–72
Affected side	
Right	58 (45.7)
Left	68 (53.5)
Bilateral	1 (0.8)
Lymphedema severity	
Grade 1	57 (44.88)
Grade 2	70 (55.12)
Observed signs (patients may have more than one)^b^	
Redness	19 (14.96)
Heat	27 (21.26)
Fibrosis	50 (39.37)
Scar	1 (0.79)
No sign	61 (48.03)
Treatment received for BCRL (patients may have more than one)^b^	
Massage	45 (35.43)
Lymphotherapy	17 (13.39)
Complementary and alternative medicine^c^	24 (18.90)
Heat therapy	18 (14.17)
Cold therapy	3 (2.36)
Exercise	1 (0.79)
No treatment	35 (27.56)

^a^Expressed as median (Quartile 1–Quartile 3); Range, other variables expressed as number (%).

^b^Patients could present with more than one sign or receive more than one treatment; therefore, percentages are not additive and may sum to > 100%.

^c^Complementary and alternative medicine included leech therapy and cupping (traditional Persian medicine). Heat and cold therapies refer to local thermal applications used by patients as self‐care remedies, not as complementary and alternative medicine.

Table 2 demonstrates the comparison of patient characteristics between the two study groups. The patients with BCRL were older at time diagnosis (49.86 vs. 46.68, *p* = 0.01), mostly living in urban areas (96.85% vs. 85.44%, *p* = 0.002) compared to those without BCRL. The percentage of employed was higher in patients without BCRL, whereas the percentage of retired was higher in those with BCRL (*p* = 0.06). The mean of BMI in patients with BCRL was more than those without BCRL (mean difference = 1.16, *p* = 0.07).

**Table 2:** Comparison of patient characteristics between patients with and without breast cancer‐related lymphedema.

Characteristics	Breast cancer‐related lymphedema		*p*
	No (*n* = 103)	Yes (*n* = 127)	
Age at diagnosis (yrs.)^a^	46.68 (8.96)	49.86 (10.33)	0.015
Marital status			
Living alone	8 (7.77)	18 (14.17)	0.127
Married	95 (92.23)	109 (85.83)	
Education level			
Illiterate	18 (17.65)	16 (12.80)	0.524
Under diploma	37 (36.27)	50 (40)	
Diploma	28 (27.45)	29 (23.20)	
Academic	19 (18.63)	30 (24)	
Occupation			
Housewife	86 (83.50)	98 (77.17)	0.064
Employed	14 (13.59)	15 (11.81)	
Retired	3 (2.91)	14 (11.02)	
Place of residence			
Urban	88 (85.44)	123 (96.85)	0.002
Rural	15 (14.56)	4 (3.15)	
Number of children			
1	18 (19.35)	16 (14.29)	0.375
2	41 (44.09)	45 (40.18)	
≥ 3	34 (36.56)	51 (45.54)	
Smoking			
No	102 (99.03)	124 (97.64)	0.630^b^
Yes	1 (0.97)	3 (2.36)	
Family history of breast cancer			
No	88 (85.44)	111 (87.40)	0.664
Yes	15 (14.56)	16 (12.60)	
Comorbidity			
No	49 (47.57)	53 (41.73)	0.375
Yes	54 (52.43)	74 (58.27)	
Body mass index (kg/m^2^)^a^	27.35 (4.20)	28.50 (5.14)	0.071

^a^Expressed as mean (SD).

^b^Exact test.

As shown in Table 3, the patients with BCRL had higher percentage of involved lymph nodes in advanced stages (e.g., for N3: 28.81% vs. 3.49%, *p* = 0.01), the percentage of ALND (e.g., for ≥ 10: 37.40% vs. 32.58%; *p* = 0.07), compared to those without BCRL. The distribution of pathology grade was significantly between the two studied groups. The percentage of Grade 3 in patients with BCRL was 36.13%, whereas this figure in another group was 31.76% (*p* = 0.004). The progesterone receptor (PR) positivity in patients with BCRL was about 14% higher than those without BCRL (75.41% vs. 61.63%, *p* = 0.03). Mastectomy surgery is more frequently performed in the case group compared with control group (34.13% vs. 21.57%, *p* = 0.03). The percentage of chemotherapy among patients with BCRL (97.64%) was higher than those without BCRL (92.23%) (*p* = 0.05). No significant differences were observed regarding radiotherapy treatment.

**Table 3:** Comparison of tumor and treatment characteristics between patients with and without breast cancer‐related lymphedema.

Characteristics	Breast cancer‐related lymphedema		*p*
	No (*n* = 103)	Yes (*n* = 127)	
Tumor size (cm)			
≤ 2	41 (47.13)	41 (34.45)	0.108
2–5	39 (44.83)	71 (59.66)	
> 5	7 (8.05)	7 (5.88)	
Lymph node			
N0	44 (51.16)	45 (38.14)	0.013
N1	28 (32.56)	29 (24.58)	
N2	11 (12.79)	34 (28.81)	
N3	3 (3.49)	10 (28.81)	
Number of axillary lymph node dissection			
≤ 5	33 (37.08)	28 (22.76)	0.070
6–9	27 (30.34)	49 (39.84)	
≥ 10	29 (32.58)	46 (37.40)	
Metastasis			
No	97 (94.17)	113 (88.98)	0.164
Yes	6 (5.83)	14 (11.02)	
Pathology grade			
1	13 (15.29)	3 (2.52)	0.004
2	45 (52.94)	73 (61.34)	
3	27 (31.76)	43 (36.13)	
Estrogen receptor			
Positive	67 (77.91)	95 (77.87)	0.995
Negative	19 (22.09)	27 (22.13)	
Progesterone receptor			
Positive	53 (61.63)	90 (73.77)	0.063
Negative	33 (38.37)	32 (26.23)	
Human epidermal growth factor Receptor 2			
Positive	33 (38.37)	50 (40.98)	0.705
Negative	53 (61.63)	72 (59.02)	
Surgery type			
Mastectomy	22 (21.57)	43 (34.13)	0.037
Lumpectomy	80 (78.42)	83 (65.87)	
Chemotherapy			
No	8 (7.77)	3 (2.36)	0.056
Yes	95 (92.23)	124 (97.64)	
Chemotherapy timing			
Neoadjuvant and Adjuvant	1 (1.05)	6 (4.84)	0.267
Only Neoadjuvant	17 (17.89)	19 (15.32)	
Only Adjuvant	77 (81.05)	99 (79.84)	
Radiotherapy			
No	3 (2.91)	6 (4.72)	0.481
Yes	100 (97.09)	121 (95.28)	
Radiotherapy timing			
Only Neoadjuvant	2 (1.98)	3 (2.48)	0.803
Only Adjuvant	99 (98.02)	118 (97.52)	

**Figure 1:**
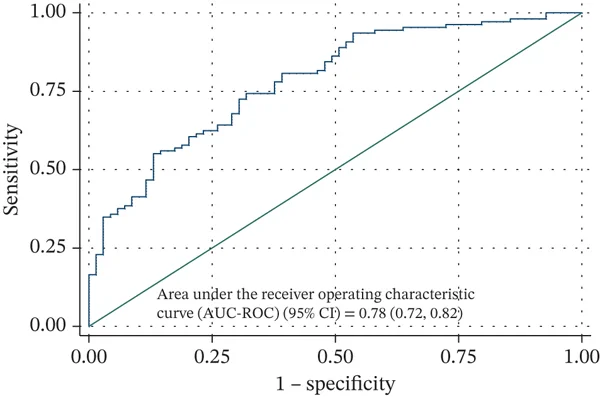
The area under the receiver operating characteristic curve (AUC‐ROC) of multivariable model for predicting breast cancer‐related lymphedema.

The multivariable analysis showed that each 1‐year increase in age at diagnosis was associated with 5% higher odds of lymphedema (OR = 1.05; 95% CI, 1.00, 1.10; *p* = 0.016). Patients residing in rural areas had 79% lower BCRL compared to those in urban areas (0.21, 0.05, 0.83; *p* = 0.026). The odds of BCRL for patients with ALND 6–9 was 2.54 times higher compared to those with ALND ≤ 5 (*p* = 0.052). Pathology Grades 2 and 3 were associated with higher odds of BCRL compared to grade 1 (OR = 7.11 and 6.53, respectively; *p* < 0.05). The odds of BCRL were approximately 2.86 times higher after mastectomy compared to lumpectomy (*p* = 0.014). PR positivity was associated with 2.56‐fold higher odds of BCRL (*p* = 0.02). Chemotherapy was also associated with increased odds of BCRL (*p* = 0.031) (Table 4). The predictive power of the multivariable model was 80% (AUC‐ROC [95% CI] = 0.78 [0.72, 0.82]) (Figure 1).

**Table 4:** Multivariable logistic regression analysis of characteristics associated with breast cancer‐related lymphedema.

Characteristics	Logistic regression		Penalized logistic regression	
	OR (95% CI)	*p*	OR (95% CI)	*p*
Age at diagnosis (yrs.)	1.05 (1.00, 1.10)	0.016	1.04 (1.01, 1.09)	0.027
Place of residence				
Urban	Reference		Reference	
Rural	0.21 (0.05, 0.83)	0.026	0.29 (0.09, 0.96)	0.042
Occupation				
Housewife	Reference		Reference	
Employed	0.82 (0.29, 2.32)	0.707	0.83 (0.32, 2.17)	0.711
Retired	3.73 (0.66, 23.47)	0.137	2.66 (0.66, 10.69)	0.169
Body mass index (kg/m^2^)	1.04 (0.96, 1.14)	0.304	1.04 (0.96, 1.13)	0.300
Lymph node				
N0	Reference		Reference	
N1	0.53 (0.22, 1.30)	0.167	0.61 (0.28, 1.37)	0.231
N2	1.60 (0.57, 4.54)	0.371	1.64 (0.65, 4.16)	0.295
N3	1.84 (0.31, 10.90)	0.498	1.50 (0.37, 6.18)	0.572
Number of axillary lymph node dissection				
≤ 5	Reference		Reference	
6–9	2.54 (0.99, 6.49)	0.052	2.15 (0.93, 5.01)	0.074
≥ 10	1.62 (0.61, 4.27)	0.327	1.57 (0.66, 3.69)	0.306
Pathology grade				
1	Reference		Reference	
2	7.11 (1.47, 34.27)	0.014	3.55 (1.03, 12.23)	0.045
3	6.53 (1.32, 32.32)	0.021	3.37 (0.95, 11.97)	0.061
Surgery type				
Mastectomy	Reference		Reference	
Lumpectomy	0.35 (0.15, 0.81)	0.014	0.38 (0.17, 0.83)	0.015
Chemotherapy				
No	Reference		Reference	
Yes	9.55 (1.23, 73.95)	0.031	4.03 (0.71, 22.69)	0.115
Progesterone receptor				
Positive	Reference		Reference	
Negative	0.39 (0.18, 0.86)	0.020	0.46 (0.22, 0.95)	0.035

Abbreviations: CI, confidence interval; OR, odds ratio.

The sensitivity analysis using penalized logistic regression showed that chemotherapy, ALND 6–9, and pathology Grade 3 were no longer statistically significant, suggesting degree of precision uncertainty in their initial estimates. However, age at diagnosis, rural residence, pathology Grade 2, mastectomy, and PR positivity remained robust, supporting their independent association with BCRL (Table 4).

## 4. Discussion

Our results showed older age at diagnosis, urban residency, greater number of ALND, higher pathology grade, mastectomy, chemotherapy, and PR positivity may be associated with BCRL.

The systematic reviews on the previous studies identified that common characteristics for predicting BCRL were BMI, radiotherapy, chemotherapy, mastectomy, and ALND [9–11, 20]. In our multivariable model, pathology grade, place of residence, ALND, PR, surgery type, chemotherapy, and age at diagnosis remained significant predictors, showing the strongest associations and making the largest contributions to the prediction. BMI, lymph node, and occupation were not significantly associated with risk of BCRL. One study in Iran showed that characteristics such as the lymph node involvement ratio, the number of removed lymph nodes, heaviness, and undergoing radiotherapy are the main and important predictors for BCRL [14]. An Australian study identified ALND, chemotherapy, radiotherapy, and BMI as significant predictors of BCRL, whereas mastectomy, age, and smoking showed no significant association [21]. A longitudinal study on the population of Latin America identified radiotherapy, obesity, seroma formation after surgery, chemotherapy infusion in the affected limb and advanced staging as significant predictors [5]. A study in China found that older age, lower income, mastectomy, postsurgical wound infection, chemotherapy, and radiotherapy significantly increased the risk of BCRL [22]. Another study on a European population identified ALND, lymph node involvement, complications following surgery, BMI, and number of dissected lymph nodes as significant predictors [23].

Our model can discriminate between female BC with and without BCRL with 78% accuracy. One study found that the application of a saturated model incorporating a combination of demographic, clinical, and lymphedema‐specific characteristics with an ensemble machine learning method yielded 91% accuracy [14]. A comprehensive review showed that the predictive power of models for predicting BCRL ranged from 68% to 91% [11]. This indicates that in order to increase the predictive ability, future studies should focus on identifying new predictors, combining biological and clinical data, and applying advanced techniques for model development and validation, such as machine learning and deep learning algorithms.

Our finding showed that PR positivity was associated with increased risk of BCRL. In the univariate analysis, there was a borderline association between PR positivity and BCRL (*p* = 0.063). Although this value did not reach the conventional threshold of 0.05, however, the PR has been included in the multivariate analysis according to a previous recommendations that variables with *p* value ≤ 0.10 or 0.20 in univariate analysis be retained for multivariable adjustment to avoid exaggeration of effect estimates [18]. Interestingly, the multivariable analysis showed a significant association for PR positivity (*p* = 0.02). However, given the borderline nature of the univariate finding, this result should be interpreted with caution and considered as hypothesis‐generating rather than conclusive. One possible explanation is that PR‐positive patients are more likely to receive adjuvant chemotherapy, which may be associated with more extensive ALND and consequently a higher risk of BCRL. Our finding is partially consistent with a recent Iranian study [24], which reported a higher odd of advanced BCRL stage among PR‐positive patients, although the association did not reach statistical significance. The directionality of both studies is similar, suggesting that PR positivity might play a role in BCRL pathogenesis, but the marginal significance underscores the need for larger, multicenter studies to confirm this relationship. The discrepancy between our findings and a previous Italian study [25], which reported a nonsignificant protective effect for PR positivity, may be attributed to differences in study design, patient characteristics, and unmeasured confounders such as hormonal therapy. Future studies specifically designed to evaluate the interplay between hormone receptor status, treatment choices, and lymphatic function are warranted to clarify this association.

Consistent with findings in previous studies [5, 26, 27], our study provides evidence regarding the impact of mastectomy and ALND on development of BCRL. However, in contrast to previous studies [28–30], our study was unable to identify radiation therapy as risk factor. The BCRL is a long‐term complication of BC treatments such as radiotherapy [31, 32]. It is likely that the association between radiotherapy and BCRL would be more easily detected if a higher percentage of patients with longer BCRL duration were included.

In this study, the relationship between BMI and BCRL was not statistically significant. The main reason is that number of patients with high BMI (e.g., > 30) was low in our study, as the mean BMI in both groups fell within the overweight category (25 < BMI < 30), and the standard deviation was less than five. The previous studies argued that BMI has a threshold effect on the risk of BCRL. For example, BMI above 30 can significantly increase the risk of BCRL [5, 21, 33].

Previous studies have found that BCRL is less likely to be diagnosed in socioeconomically disadvantaged areas [21, 34]. In contrast to previous studies, our study showed that the percentage of BCRL in patients residing in urban areas is higher than that of those in rural areas. It should be noted that accessibility to screening, care and diagnosis services is greater in urban areas, which increases the likelihood of detecting outcomes such as BCRL. Therefore, potential biases including surveillance bias, detection bias, and volunteer bias should be carefully considered when interpreting these results.

Our findings were consistent with previous Iranian studies. A study in Shiraz, Southern Iran, reported that modified radical mastectomy, number of excised lymph nodes, radiotherapy, and BMI ≥ 25 were significant predictors of BCRL [35]. Another study on patients from Tehran (Central Iran) and Mashhad (Northeast Iran) found that each unit increase in BMI and each additional involved lymph node significantly increased BCRL risk [36]. Furthermore, a multicenter study provided evidence regarding the potential impact of mastectomy, radiotherapy, and supraclavicular radiation on BCRL, further supporting the role of treatment intensity [37]. Our findings on patient, tumor, and treatment characteristics were relatively consistent with these reports. However, our study extends the existing literature by providing a risk profile for Western Iran, highlighting urban/rural disparities, and identifying PR positivity as a potential biological predictor of BCRL.

Although our sensitivity analysis did not confirm the statistical significance of some variables such as chemotherapy, ALND 6–9, and pathology Grade 3, these factors should not be ignored as clinically unimportant. It is crucial to distinguish between statistical significance and clinical importance, particularly in the context of limited sample sizes. The wide CIs and unstable estimates observed in our study likely reflect insufficient statistical power rather than a true absence of effect. Therefore, although these findings should be interpreted cautiously, the potential clinical relevance of these risk factors remains, and their role should be further investigated in larger prospective cohorts with adequate power to detect modest but clinically meaningful associations.

There were several limitations that should be considered. First, modeling without validation adds nothing; therefore, it is still necessary to externally validate the developed model using independent datasets. Second, BCRL was measured in dichotomous scale. The categorization of variable can lead to loss of information. Considering other forms of the BCRL, such as its severity or volume, can reveal more accurately and precisely the effects of tumor, treatment and patient risk factors on BCRL. These two limitations have not been addressed because of limited sample size. For example, there was not enough data to apply *k*‐fold cross‐validation to assess the model′s validity. Third, there was limited precision for some risk factors, such as chemotherapy, ALND 6–9, and pathology Grade 3, likely due to sparse data bias. Therefore, larger prospective studies are warranted to provide more precise estimates of these associations. Fourth, the multivariable prediction model for BCRL still needs to be enhanced by incorporating additional risk factors such as BC diagnosis (e.g., ductal carcinoma in situ and invasive lobular carcinoma), mammographic breast density, treatment features (e.g., hormones therapy and number of chemotherapy sessions). Fifth, missing data for some characteristics may have introduced bias, although the small proportion and random nature of missingness (due to incomplete records) suggest minimal impact on the findings. Finally, although both centers for selection of cases and controls are affiliated with Hamadan University of Medical Sciences, serve the same geographic catchment area, and utilized consistent eligibility criteria, the degree of selection bias should still be considered. However, we believe that the potential impact of this bias on our results might be limited. Although we observed differences in age, residency, and occupation—which were adjusted for in multivariable analysis—there were no significant differences in other baseline characteristics such as marital status, education, family history, and comorbidity, supporting the overall comparability of the groups. Additionally, any residual selection bias would likely attenuate the observed associations (i.e., bias towards the null) because controls recruited from the radiology center are likely to have a higher prevalence of aggressive treatments (e.g., ALND, mastectomy, chemotherapy) compared to the general population of BC survivors, as evidenced by the high proportions of controls who had received chemotherapy (92.23%), radiotherapy (97.09%), and ALND with ≥ 10 nodes dissected (32.58%). This would reduce the contrast between cases and controls, leading to an underestimation of the true associations. Therefore, any significant associations we observed are likely conservative estimates of the true effects. Future population‐based or prospective studies are warranted to confirm these results.

## 5. Conclusion

Our findings confirm that some well‐known treatment‐related factors remain important predictors of BCRL. However, the results observed in the current study highlight several clinical implications for the Iranian healthcare system. The urban/rural disparity in BCRL suggests the presence of surveillance/detection bias, implying that the true burden of BCRL in rural areas may be underestimated. Consequently, this calls for enhanced outreach and screening programs in underserved regions. The independent role of PR positivity, alongside pathological grade, highlights that tumor biology—not just treatment intensity—may contribute to lymphatic vulnerability. This finding encourages future research into hormonal influences on fluid homeostasis and tissue fibrosis. Finally, the developed risk model provides a simple, accessible tool for clinicians in Western Iran to identify high‐risk patients (older age, high‐grade tumors, mastectomy, and PR‐positive) who would benefit most from prospective surveillance and early physiotherapy interventions, ultimately reducing the morbidity and healthcare costs associated with chronic lymphedema.

## Author Contributions

Soulmaz Rahbar and Erfan Ayubi conceptualized and supervised the study. Soulmaz Rahbar, Hojjat Radinmehr, Mohammad Reza Asadi, and Abdolazim Sedighi Pashaki participated in data collection and data curation. Soulmaz Rahbar, Tayebeh Roghani, Azade Tabatabaei, Abdolazim Sedighi Pashaki, and Erfan Ayubi contributed to methodology development and data interpretation. Soulmaz Rahbar and Erfan Ayubi performed the statistical analysis. Soulmaz Rahbar and Erfan Ayubi drafted the manuscript.

## Funding

This work was supported by the Hamadan University of Medical Sciences, 10.13039/501100004697, 140303292698.

## Disclosure

All authors critically reviewed and revised the manuscript, approved the final version, and agreed to be accountable for all aspects of the work.

## Ethics Statement

The study was approved by the Research Ethics Committee of the Hamadan University of Medical Sciences (IR.UMSHA.REC.1403.192).

## Conflicts of Interest

The authors declare no conflicts of interest.

## Data Availability

The data that support the findings of this study are available from the corresponding author upon reasonable request.
